# Helical tomotherapy craniospinal irradiation in primary brain tumours: Toxicities and outcomes in a peadiatric and adult population

**DOI:** 10.1016/j.ctro.2024.100777

**Published:** 2024-04-06

**Authors:** Julie Savagner, Anne Ducassou, Bastien Cabarrou, Gregory Hangard, Marion Gambart, Anne-Isabelle Bertozzi, Eloise Baudou, Sergio Boetto, Delphine Larrieu, Anne Laprie

**Affiliations:** a Department of Paediatric Neurology, Children’s Hospital of Toulouse, 330 Avenue de Grande Bretagne, 31300 Toulouse, France; b Department of Radiation Oncology, Toulouse Cancer Institute (IUCT), 1 avenue Irene Joliot-Curie, 31100 Toulouse, France; c Department of Biostatistics, Toulouse Cancer Institute (IUCT), 1 avenue Irene Joliot-Curie, 31100 Toulouse, France; d Department of Paediatric Oncology, Children’s Hospital of Toulouse, 330 Avenue de Grande Bretagne, 31300 Toulouse, France; e Department of Neurosurgery, Toulouse University Hospital, Pierre-Paul Riquet Hospital, Place du Docteur Baylac, Toulouse, France; f Department of Oncology, Toulouse Cancer Institute (IUCT), 1 avenue Irene Joliot-Curie, 31100 Toulouse, France

**Keywords:** Craniospinal irradiation, Helical tomotherapy, Brain tumour, Medulloblastoma, Pediatric cancer, Late effects

## Abstract

**Objective:**

As craniospinal irradiation (CSI) is delivered more frequently by helical tomotherapy (HT) with few reports about late effects, we analysed all patients treated in our centre over an 11-year period.

**Methods and materials:**

Our study included all patients that underwent CSI by HT, between September 2009 and January 2020, in the Department of Radiation Oncology of the Toulouse Cancer Institute. Acute radiotherapy toxicities were reported and medium- to long-term outcomes analysed.

**Results:**

Among the 79 patients included, 70.9 % were younger than 18 years at diagnosis, the median age was 13 (range: 1–52) at the time of radiation therapy, 67.1 % of patients had medulloblastoma. Half of them (49.4 %) had a metastatic disease at diagnosis. The median dose of CSI was 36 Gy (range, 18–36). Seventy-seven patients received a radiation boost to the original location of the primary tumour (97.5 %), 32 patients also received a boost to their metastatic sites (40.5 %). Median follow-up was 55.5 months (95 %CI = [41.2; 71.8]). The 3-year event-free survival rate was 66.3 % (95 %CI = [54.2; 75.9]). Most patients presented with acute haematological toxicities during CSI (85.9 %), predominantly severe thrombocytopenia (39.7 %). Among the 64 patients assessed for medium- and long-term outcomes, 52 survived and 47 were alive and disease-free at the latest follow-up visit on record. There were 3.8 % secondary tumours: two meningiomas and one diffuse intrinsic pontine glioma. Adult and paediatric patients respectively presented with secondary cataract (4.3 % vs 22.0 %), persistent hearing disorders (26.1 % vs 29.3 %), pulmonary or cardiac late effects (4.3 % vs 2.4 %), hormonal pituitary gland deficiencies (30.0 % vs 56.8 %) and psycho-cognitive disorders (56.5 % vs 53.7 %).

**Conclusion:**

CSI dispensed by HT, did not result in any additional acute or late toxicities when compared to 3D-CSI. There was no increase in the secondary tumour rate compared to that reported in the literature.

## Highlights

• Helical tomotherapy is effective for craniospinal irradiation of several CNS tumours.
• HT-CSI is delivered more often but there are few data about late effects.
• Tomotherapy offers technical advantages when compared to other radiation techniques.
• HT-CSI provides good organ-at-risk sparing with no additional acute or late toxicities.

## Introduction

Several central nervous system (CNS) malignancies, such as medulloblastomas, are prone to cerebrospinal fluid dissemination. The initial clinical management predominantly includes: maximal surgical resection followed by chemotherapy and radiation therapy of the whole brain and the entire craniospinal axis with an additional boost to the primary tumour. As for other malignant tumours, their management may require craniospinal irradiation (CSI) in case of leptomeningeal dissemination, with a boost to the primary site. Radiation doses are determined based on tumour type and risk profile. Adequate dose coverage of the whole CNS is essential to prevent recurrence of the disease. However, delivering a uniform dose throughout such a large volume is very technically very challenging, as poor dose distribution may increase the risk of treatment failure, and high radiation doses to organs at risk (OARs) may cause acute and late toxicities. Irradiation of the entire craniospinal axis was previously achieved with conventional 3D-conformal radiotherapy (3D-CRT); but this technique had many disadvantages, principally heterogeneous dose coverage of the target due to field gaps and junctions [1]. Several published reports have highlighted the dosimetry benefits of Intensity-Modulated Radiation Therapy (IMRT)–based technique, such as Helical Tomotherapy (HT) to deliver CSI [2], [3], [4]. HT improves the dose coverage of the target enabling large volumes to be irradiated continuously and homogeneously [5], without gaps or junctions [6]; and reducing the dose to selected critical organs [7], [8]. HT also allows patients to be placed in the supine position thus improving patient comfort and treatment reproducibility. However, these benefits of HT-IMRT are generally achieved at the cost of greater volume of normal tissue being irradiated with low doses [9], [10]. The deleterious effects of radiation therapy are volume-, dose- and age-dependent; resulting from damages to healthy tissues within the irradiated volume [11], [12]. Although HT reduces the occurrence of acute adverse events, many patients still experience haematological toxicities, particularly if their treatment included chemotherapy before radiation therapy. Survivors often suffer from treatment-induced sequelae which decrease the quality of life. The common late-effects after CSI, particularly in children, include sensory, neuroendocrine, and neurocognitive disorders [13]. Secondary tumours are less common, but represent an important cause of late death, with meningioma being the most frequently observed benign tumour and high-grade glioma representing almost half of secondary malignancies [14], [15]. The aim of our study was to evaluate the occurrence of acute, medium and late adverse events in 79 patients with primary brain tumours treated with HT-CSI.

## Materials & methods

All patients that underwent CSI with HT, in the Department of Radiation Oncology of the Toulouse-IUCT, between September 2009 and January 2020, were retrospectively analysed. The data gathering period ended in July 2021. Data was based on the last examination of all the patients alive or lost to follow-up at last collection. Patients or guardians, had provided written informed consent, including medium to long term follow-up. The study was approved by local institutional boards.

### Radiotherapy planning

HT is based on the combined principles of a linear accelerator and a computed tomography scanner. CT images for planning were acquired in the supine position using an immobilisation and positioning system, devised for each individual patient. The craniospinal clinical target volume (CTV) was defined as whole brain and spinal cord, from C1 to S2-S3. Planning target volume (PTV) was defined as the CTV with a uniform margin of 0.5 to 1 cm depending on age, in accordance with international guidelines. CSI doses were prescribed as recommended for each tumour type and defined risk groups [16], [17], [18], from 18 to 36 Gy [19], [20], [21].

In most cases, an additional boost to the original tumour bed was recommended; with a total of 40 to 54 Gy depending on tumour type, target volume and site. Cerebral or spinal metastases were focally irradiated, with 45 to 54 Gy for spinal metastatic sites located above or below L2, respectively. All dosimetry results, for both target and OAR volumes, were evaluated from dose-volume histograms. All patients received prophylactic Ondansetron before undergoing CSI and had at least one weekly complete blood count.

### Assessment of adverse events

All patients were evaluated for acute toxicities at the time of the CSI and for medium- and long-term outcomes from 6 months after completion of radiotherapy. Side effects were recorded according to the CTCAE guidelines version 5.0. Common acute toxicities such as fatigue, anorexia, alopecia, nausea, vomiting, or neurotoxicity were not evaluated.

Only patients that did not relapse or progress and that were alive 6 months after completing the CSI were assessed for medium- and long-term toxicities. This follow-up examination focused on disease status (relapse, progression, remission) and occurrence of any adverse effects of therapy. Adverse events and sequelae were evaluated relative to craniospinal and related OAR volumes. Reported side effects included: endocrine, neurological, neurocognitive, respiratory, cardiac, musculoskeletal, ophthalmologic and hearing disorders.

### Statistical analyses

Categorical variables are summarised as frequencies and percentages, and continuous variables as medians and ranges. Associations between radiotherapy doses and the different toxicities were assessed using the Chi-squared or Fisher’s exact test for categorical variables and the Mann-Whitney test for continuous variables. Event-free survival (EFS) was defined as the time interval between the CSI start date and the date of relapse or progression or death from any cause. Overall survival (OS) was defined as the interval between the CSI start date and the date of death from any cause. Survival rates were estimated using the Kaplan-Meier method with their 95 % confidence intervals (95 %CI). All statistical tests were two-sided and a p-value < 0.05 was considered statistically significant. Statistical analyses were carried out using the STATA v16 (StataCorp, College Station, TX, USA) software.

## Results

A total of 79 patients with histologically confirmed brain tumours and treated with HT-CSI were analysed. Median age at diagnosis and at the beginning of CSI was 11 (range, 0–52) and 13 years (1–52), respectively. Fifty-six patients (70.9 %) were less than 18 years of age at diagnosis. Almost half of the patients (49.4 %) had metastatic diseases at diagnosis. CSI was a first-line treatment for most patients (83.5 %). CSI was recommended as neuro-meningeal prophylaxis in 27 localised medulloblastoma cases (34.2 %). The main patient and tumour characteristics are listed in Table 1.

**Table 1:** Patient, tumour and treatment characteristics.

**Characteristics**	Value*
Median age at diagnosis in years (range)	11 (0–52)
Median age at beginning of CSI in years (range)^b^	13 (1–52)
Age ≤ 3 years at diagnosis	11 (13.9%)
Age 3–5 years at diagnosis	7 (8.9%)
Age 6–17 years at diagnosis	38 (48.1%)
Age ≥ 18 years at diagnosis	23 (29.1%)
Female	26 (32.9%)
Male	53 (67.1%)
Posterior fossa Tumour	56 (70.9%)
Supra-tentorial Tumour	20 (25.3%)
Spinal Tumour	3 (3.8%)
Medulloblastoma	53 (67.1%)
Germ cell Tumour	8 (10.1%)
Choroid plexus Tumour	5 (6.3%)
Ependymal Tumour	4 (5.1%)
Metastatic stage	39 (49.4%)
Chemotherapy	62 (78.5%)
Pre-radiotherapy	36 (45.6%)
Pre-radiotherapy HDCT^c^	26 (32.9%)
HDCT Thiotepa	20 (25.3%)
Concurrent	5 (6.3%)
Adjuvant	17 (21.5%)
Maintenance Temozolomide	19 (24.1%)
Gross total resection/near total resection	36 (45.6%)
Subtotal resection	41 (51.9%)
Biopsy alone	2 (2.5%)
CSI dose in Gy (range)	36 (18–36)
Total dose to tumour bed/primary tumour in Gy (range)	54 (26–68)
Boost to metastatic sites	32 (40.5%)
Treated twice daily	4 (5.1%)
**Total**	79 (100%)

* The values listed in the table give the number of patients (n) followed by the percent of patients (%), unless otherwise specified.

b CSI: Craniospinal Irradiation

c HDCT: High Dose Chemotherapy

Seventy patients (88.6 %) had primary tumour surgery, with complete resection achieved in 51.4 % of cases. Chemotherapy regimens were administered according to cancer specific protocols: 78.5 % of patients received chemotherapy in neoadjuvant, adjuvant, or concomitant settings; 45.6 % were treated with neoadjuvant Carboplatin-Etoposide and 6.3 % underwent concomitant chemotherapy during radiotherapy (weekly Vincristine in most cases). High-dose chemotherapy (HDCT) followed by autologous peripheral stem cell rescue prior to CSI was achieved in 32.9 % patients, mostly with high dose Thiotepa (76.9 %). The median time interval between HDCT and the start of CSI was 7.5 weeks (range, 6–72). After completing CSI, 21.5 % of patients had adjuvant intravenous chemotherapy, and 24.1 % had oral Temozolomide as maintenance chemotherapy.

The median time interval between diagnosis and starting of CSI was 4.7 months (range, 0.8–227.8). Median duration of radiotherapy (including both CSI and boost) was 6.1 weeks (range, 1.0–8.0). Median dose of CSI was 36 Gy (range, 18–36). Median number of fractions for CSI was 20 (range, 10–36). The boost irradiation was realised with photon-VMAT in 97.5 % of cases, 2.5 % were treated for medulloblastoma with proton-therapy on their primary tumour site. A radiation boost to one or more metastatic site was added in 32 patients (40.5 %), of which 31.2 % received 54 Gy to supratentorial sites, 43.8 % received 30 to 45 Gy to medullar disseminations, and both supratentorial and medullar sites were irradiated in 25.0 %. Details of prescribed HT-CSI doses and fractionations are listed in Table 2.

**Table 2:** Radiotherapy characteristics of craniospinal irradiation (CSI), primary tumour boost doses, and fractionations.

**CSI**		**Daily fractionation**		**Boost to primary tumour site**	
**CSI dose**	**n (%)**	**Daily dose**	**n**	**Boost dose (total dose)**	**n**
**18 Gy**	5 (6.3 %)	1.8 Gy	4	+ 36 Gy (54 Gy)	4
		1.6 Gy	1	+ 27 Gy (45 Gy)	1
**23.4 Gy**	16 (20.3 %)	1.6 Gy	16	+ 30.6 Gy (54 Gy)	16
**24 Gy**	5 (6.3 %)	1.6 Gy	5	+16 Gy (40 Gy)	4
				+30 Gy (54 Gy)	1
**30 Gy**	3 (3.8 %)	1.6 Gy	3	+ 24 Gy (54 Gy)	3
**36 Gy**	50 (63.6 %)	1 Gy BID*	4	No boost	2
				+ 9 Gy (45 Gy)	2
		1.8 Gy	46	+18 Gy (54 Gy)	38
				+24 Gy (60 Gy)	8
**Total**	**79 (100 %)**				

* 1 Gy twice a day (BID) radiation therapy: Hyper-fractionated radiotherapy with a smaller dose per fraction (1 Gy), with radiotherapy treatment administered twice each day, usually 6–8 h apart.

The median follow-up time was 55.5 months (95 %CI = [41.2;71.8]). Thirty-two patients (40.5 %) relapsed or progressed after completing treatments, this occurred within 6 months of completing CSI in 8 of them (25.0 %). The median times from diagnosis to relapse time interval was 34.47 months (range, 8–132). At the end of the follow-up, 69.6 % of patients survived and 59.5 % were alive and disease-free. The 3-year EFS and OS were 66.3 % (95 %CI = [54.2;75.9]) and 80.7 % (95 %CI = [69.4;88.2]), respectively. There was no toxic death related to CSI.

**Fig. 1:**
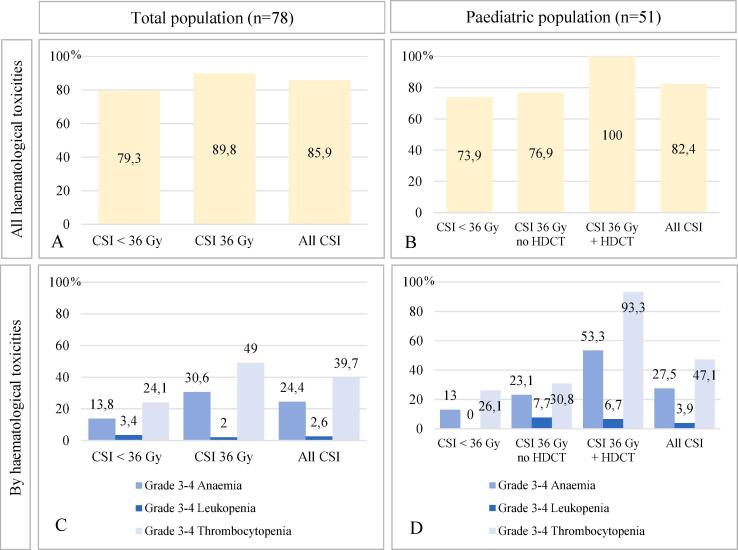
Acute haematological toxicities by CSI dose (<36 Gy vs 36 Gy CSI) in the overall population (n = 78), and according to treatment intensification with High Dose Chemotherapy (HDCT) in patients < 18 years of age (n = 51). (A) and (B) represent all acute haematological toxicities by CSI dose (and HDCT in children), with a significant difference in the paediatric population (p = 0.06, vs p = 0.31 in the overall population). C and D represent different acute haematological toxicities (anaemia, leukopenia and thrombocytopenia) according to CSI dose (and HDCT in children). (C) represents the adult population, (D) represents the paediatric population. There is a statistically significant difference between CSI doses for Gradev3-4 thrombocytopenia in the overall population (p = 0.03) and for Grade 3–4 anaemia and thrombocytopenia in the paediatric population (respectively, p = 0.03 and p < 0.01).

The original CSI schedule was modified in 7 patients (9.0 %) due to severe side effects. CSI was stopped, then the boost was delivered and the CSI was subsequently restarted in 6.3 % of patients who ended up receiving the complete radiation that was initially planned; radiotherapy was discontinued in 2 adult metastatic patients (2.5 %). Introduction or escalation of corticosteroids during radiotherapy was necessary in 93.6 % of cases. Most patients had acute haematological toxicities (85.9 %), including: leukopenia (any grade: 44.9 %, grade III-IV: 2.6 %), anaemia (any grade: 46.2 %, grade III-IV: 24.4 %), and thrombocytopenia (any grade: 85.9 %, grade III-IV: 39.7 %). Severe thrombocytopenia was significantly more frequent in the 36 Gy CSI group (p = 0.03). Most patients with grade III-IV thrombocytopenia younger than 18 years of age (77.4 %) and had received HDCT (64.5 %). Data on acute haematological events in different CSI groups are provided in Fig. 1.

In the 6 months after CSI, 15 patients had early events (relapse, progression or death). The remaining 64 patients were available for long-term follow-up (81.0 %). The main reported organ toxicities and their related OAR doses are detailed in Table 3. Specific toxicities of the paediatric population are given in Table 4.

**Table 3:** Dose distribution to the organs at risk and main related toxicities.

**OARs**	**Median Doses in Gy** (range)		**Related medium- and long-term toxicities (% of assessed patients)**			
	Dmax*	Dmean^**^	**Toxicity**	**Total (n = 64)**	**< 18 y.o.** (n = 41)	**≥18 y.o.** (n = 23)
Brain	55.6 (25.4–102.1)	38.9 (23.7–48.5)	Psycho-cognitive	54.7%	53.7%	56.5%
Brainstem	54.5 (18.3–105.6)	49.7 (7.0–64.9)	Non-assessed			
Chiasma	41.8 (15.4–55.3)		*See Eye section*			
Cochlea - Left	42.9 (10.0–51.7)	36.5 (13.9–46.9)	Hearing disorders	28.1%	29.3%	26.1%
Cochlea - Right	41.7 (19.3–52.3)	36.2 (11.9–44.4)				
Eye - Left		27.6 (5.3–47.1)	Eye disorders (excluding cataract)	28.1%	39.0%	8.7%
Eye - Right		(27.1 (4.4–44.8)				
Gonads		0.3 (0.1–8.0)	Non-assessed			
Heart		7.8 (3.4–15.7)	Cardiac disorders	3.1%	2.4%	4.3%
Hippocampus - Left	50.5 (23.8–83.0)	45.0 (18.4–61.0)	Memory disorders	35.2%	35.1%	35.0%
Hippocampus - Right	52.0 (23.8–82.0)	45.2 (23.2–60.2)	Attention deficiency	26.3%	29.7%	20.0%
Kidney - Left		6.1 (2.4–12.3)	No reported toxicity			
Kidney - Right		6.0 (2.1–12.1)				
Liver		7.4 (2.0–13.1)	No reported toxicity			
Lens - Left		21.3 (5.8–40.5)	Secondary cataract	15.6%	22.0%	4.3%
Lens - Right		20.4 (5.9–36.7)				
Lungs		7.1 (1.4–13.1)	Respiratory disorders	3.10 %	0.00 %	8.70 %
Oesophagus	30.6 (14.0–42.2)		Non-assessed			
Optic Nerve - Left	39.2 (13.3–55.0)		*See Eye section*			
Optic Nerve - Right	38.9 (12.2–54.3)					
Parotid gland - Left		15.0 (1.8–28.4)	Non-assessed			
Parotid gland - Right		15.9 (2.1–28.9)				
Pituitary gland	41.7 (18.1–66.2)	39.3 (10.8–57.9)	Hypopituitarism	47.4%	56.8%	30.0%
			*GH deficiency*	*31.8%*	*43.2%*	*10.0%*
Thyroid gland		13.5 (3.8–25.5)	Hypothyroidism^+^	26.3%	32.4%	15.0%

Some elevated doses can be explained by the fact that prior irradiations on related OARs were included.

* Dmax = maximal dose, ^**^Dmean = mean dose, ^+^Hypothyroidism included mixed central & peripheral defect in most patients.

**Table 4:** Main OAR-related medium and long-term toxicities in the paediatric population (n = 38). Three children who had prior CNS radiation therapy were excluded from the analysis. We assessed 38 children for most toxicities, but only 35 for endocrine disorders (n = 35) because data was missing for 3 patients.

**Main medium and long-term toxicities in children**		
**Toxicity**	**n**	**%**
**Endocrine disorders**	**23**	**65.7 %**
Hypopituitarism	20	57.1%
Isolated GH deficiency	15	42.9%
Hypothyroidism*	11	31.4%
**Eye disorders**	**23**	**60.5%**
Secondary cataract	15	39.5%
Other eye disorders^**^	8	21.1%
**Psycho-cognitive**	**20**	**52.6%**
Memory disorders	14	36.8%
Attention deficit	11	28.9%
Psychomotor retardation	7	18.4%
Executive dysfunction	5	13.2%
Apraxia	4	10.5%
Agnosia	3	7.9%
Communication disorders	3	7.9%
**Hearing disorders**^**+**^	**11**	**28.90 %**
**Cardiac disorders**^**++**^	**1**	**2.6%**

* Hypothyroidism of both central and peripheral origin, ^**^Such as visual field loss, oculomotor disorders or decreased visual acuity, ^+^ Such as hearing loss, and vestibular disorders, ^++^ Such as heart failure and cardiac rhythm disorders.

Overall, 45 patients (60.0 % of assessed patients; mean age: 9.4 years, range: 1.6–34.5) presented substantial endocrine toxicities, including 46.9 % with hypopituitarism. The most frequently reported endocrine defect was growth hormone (GH) deficiency (31.2 %) but this was not significantly associated with higher mean doses to the pituitary gland (GH deficient patients received a median dose of 41.9 Gy, vs 39.1 Gy for patients with no GH deficiency; p = 0.10). Only 6 patients received a pituitary gland mean dose of less than 16 Gy; none of these patients developed a GH deficiency. Hypothyroidism was reported in 29.7 % of patients, mostly predominantly peripheral hypothyroidism, potentially due to the irradiation of the thyroid.

Reported neurocognitive impairment (54.7 %; mean age: 10.2 years, range: 1.6–27) included: memory disorders (34.4 %), attention deficiency (26.6 %), psychomotor retardation (15.6 %), executive dysfunction (15.6 %), apraxia (10.9 %), agnosia (9.4 %), and communication disorders (6.3 %). Neurocognitive impairment was not associated with higher doses to the brain or to the hypothalamus. Mild to moderate heart failure and respiratory insufficiency were reported in 3.1 % of patients. Hearing loss occurred in 28.1 % of cases, with 6.2 % of patients requiring a hearing aid or an intervention for a severe (grade III) condition. Symptomatic cataracts affected 15.6 % of patients, with 9.4 % undergoing cataract surgery. No significant associations were detected when these disorders were analysed as a function of the mean CSI doses received by the cochlea (p = 0.14) or the lenses (p = 0.31).

Focusing on the paediatric population, there were no significant differences between the CSI dose groups in terms of sensory, endocrine, neurological and cognitive outcomes. Young patients who did not receive HDCT reported fewer anxiety and depressive disorders (21.4 % vs 0.0 %, p = 0.04). Long-term toxicities occurred more frequently in paediatric patients, with the exception of psychocognitive and cardiovascular disorders.

A biopsy confirmed, partial asymptomatic osteoradionecrosis of a lumbar vertebra occurred in one patient who had been treated for a metastatic medulloblastoma 12 years earlier, craniospinal dose was 36 Gy with no spinal boost. Maximal and mean doses to the vertebra were 37.7 and 34.2 Gy.

Three young patients (4.7 %) developed secondary tumours. Two of these tumours were benign grade I meningiomas. Both occurred in supratentorial regions that received boosts, of 54.3 Gy and 55.6 Gy, respectively 7 and 8 years after CSI. They were alive without disease at the end of follow up. The third tumour was a malignant diffuse intrinsic pontine glioma (grade IV with no histone mutation) arising 6 years and 11 months after CSI in a patient previously treated for a metastatic anaplastic medulloblastoma at the age of 18 months. She had received CSI of 18 Gy, and additional boosts of 27 Gy to the primary site (total 45 Gy) and 12 Gy to a supratentorial metastasis. Maximal and mean doses to the brainstem were respectively 46.7 and 41.1 Gy. She died 8 months after the diagnosis of the secondary brainstem glioma.

Prior CNS radiotherapy was not significantly associated with higher acute or late toxicities. Seven patients (8.9 %) had previously received CNS radiotherapy and data from 4 of these patients was available for long-term follow-up assessment. None of these patients developed had any secondary malignancies.

## Discussion

Previous studies have confirmed the advantages of HT, over 3D-CRT or IMRT, as it allows to irradiate the entire neuroaxis in one single volume [22], and delivers homogeneous radiation, thus avoiding over- or under-dosing [23], [24]. HT has better target volume dose distribution and homogeneity compared to conventional or intensity-modulated techniques [25], [26], and also reduces the dose delivered to OAR [27], [28]. Even in paediatric patients, HT-CSI has shown to have tolerable normal organ doses [29]. However, there are currently very few formal dosimetry comparisons for individual CSI techniques using the same patient dataset [30].

Haematological toxicities in patients undergoing CSI are frequent and well described in the literature [29], [31]. We identified a greater proportion of grade III or IV toxicities, compared to existing reports (5 to 42 % severe thrombocytopenia), which is potentially due to our wider range of CSI patients and the higher mean CSI doses delivered [32], [33], [34]. One out of 3 of our patients (32.9 %) underwent HDCT prior to CSI, leading to more bone marrow toxicities. The onset of haematological toxicities during CSI appeared to be related to the radiation dose, specifically in the case of severe toxicities, but did not reach significance when comparing CSI groups except for severe thrombocytopenia.

All potentially irradiated organs resulted in medium- and long-term toxicities. Although several CNS disease treatments have resulted in improved survival, there are growing concerns about long-term radiotherapy-related side effects, particularly endocrine dysfunction, hearing impairment, development of cataract, neurocognitive decline and secondary malignancies [35], [36]. GH deficiency is the most common endocrine dysfunction, predominantly arising after the first year following radiotherapy [37]. Our GH deficiency rate (31.2 %) was inferior to that generally reported in the literature (from 39 % to 94 % in children), this is potentially due to both lower toxicity and the fact that fewer studies have investigated older patients [38], [39], [40]. Auditory toxicity concerned 28.1 % of patients, similar to the reported rate of 25 to 39 % [41], [42], [43]. Irradiation of the whole brain and specifically the hippocampi may lead to neurocognitive toxicity. Previous studies showed that about 70 % of long-term survivors developed some form of neurocognitive dysfunction within, a minimum, 15-year follow-up [44], [45], [46]. This is higher than our 54.7 % rate of patients with cognitive disorders, possibly related to the lack of neuropsychological assessment of our study. We did not find any significant associations between cognitive disorders and radiation doses to the brain or the hippocampi. Several studies have shown that the decline in cognitive activity can be attributed to multiple additional risk factors, such as the radiation dose [47], but also tumour-induced hydrocephaly, young age at diagnosis, hearing loss, or post-operative posterior fossa syndrome [48], [49], [50]. Pre-existing neurological deficiencies frequently were frequent prior to undergoing radiation therapy, this is predominantly due to the tumour itself and/or the surgical resection.

Secondary neoplasms are a well-established long-term adverse effect of radiation therapy [51], but there are limited data regarding their incidence and location in terms of the radiation field after CSI. The cumulative incidence of secondary neoplasms 10 years after CSI is reported to be 6.1 % for all secondary cancers, with 68 % of these cancers being malignant [14]. The risk of secondary cancers depends on the length of the follow-up, and ranges from 1.7 % to 13 % depending in the study. HT techniques might lead to an increased risk of secondary tumours outside of the CNS with a larger spread of low-dose radiation compared to 3D-CRT [15]. In our report, considering assessable patients only, 3 (4.7 %) had secondary tumours, of which one (1.3 %) was malignant. The low rate of secondary malignancies in our study may be explained by the shorter follow-up period (4.6 years vs 5 to 10 years in most publications) [52], [53].

The current study suffers several limitations: the low number of patients that could be assessed for medium- and long-term adverse events, the heterogeneity of the population with two distinct main indications for CSI (as a first-line treatment for embryonal tumours, and second-line treatment for disseminated tumours or distance relapses), and the retrospective nature of assessing individual organ-related toxicities. The impact of radiotherapy on cognition was also difficult to evaluate as there were not pre-radiotherapy cognitive evaluations. There was no systematic psychological assessment. Moreover, the follow-up, which extended to a median of 55 months, would need to be regularly updated to allow a more comprehensive analysis of long-term outcomes, including secondary malignancies.

Most long-term events depend on the volume and dose of the radiotherapy [54]. Future perspectives include an adaptation of CSI radiation doses, based on the molecular profile of the individual disease [55]. Even though late effects are also of great concern for metastatic patients, their worse prognosis does not allow a dose reduction, as the risk/benefit ratio imposes intensified chemotherapy which in turn results in increased radiation toxicity. Advanced photons techniques such as tomotherapy can reduce radiation doses delivered to OARs outside of the target volume and greatly improve treatment quality [56]. However, these techniques also increase the volume of low-dose irradiation delivered to healthy tissues, which needs to be taken into account when considering long-term issues [57].

Proton therapy could be the ideal tool to reduce toxicity due to non-target doses, with a potential beneficial effect on preservation of cognitive function, specifically for boosts, which may in turn limit the diffuse dose received by supratentorial structures and thyroid [40], [58], [59], [60]. Access to proton therapy was very limited at the time of the study, with only a few of our patients treated by proton for the boost irradiation to their primary tumour site. Recent studies comparing the late effects of proton and photon CSI have not found a difference in the incidence of secondary tumours at the 10-year follow-up [52].

CSI remains an integral part of CNS malignancies treatment. Helical tomotherapy has many advantages in terms of homogeneous target volume coverage and patient comfort, but entails a different dose delivery to OARs compared to conventional 3D techniques. Our current study did not find supportive evidence for any additional acute or late toxicities due to HT, specifically no increases in the secondary cancer rate.

## Conflict of interest

All authors declare that there is no conflict of interest.

## Declaration of competing interest

The authors declare that they have no known competing financial interests or personal relationships that could have appeared to influence the work reported in this paper.
