# Supplementary material for: Helical tomotherapy craniospinal irradiation in primary brain tumours: Toxicities and outcomes in a peadiatric and adult population
Source: Clin Transl Radiat Oncol. 2024 Apr 6;46:100777. doi: 10.1016/j.ctro.2024.100777 (PMC11019098; doi:10.1016/j.ctro.2024.100777)

SUPPLEMENTARY MATERIALS:

Supplementary Figure: Kaplan-Meier Survival analysis of the overall population. A: Event-Free Survival (EFS), and B: Overall Survival (OS). The 3-year EFS and OS were 66.3% (95%CI= [54.2;75.9]) and 80.7% (95%CI= [69.4;88.2]), respectively.

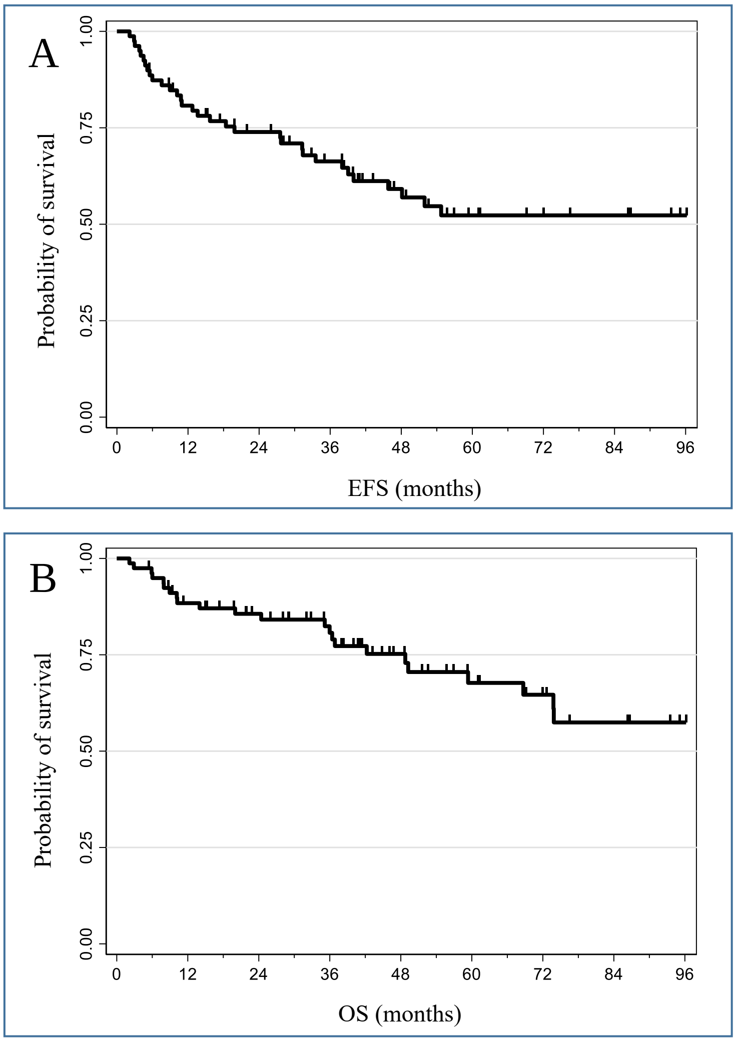

Supplement: Supplementary data 1 [file mmc1.pdf]
