# Supplementary material for: Helical tomotherapy craniospinal irradiation in primary brain tumours: Toxicities and outcomes in a peadiatric and adult population
Source: Clin Transl Radiat Oncol. 2024 Apr 6;46:100777. doi: 10.1016/j.ctro.2024.100777 (PMC11019098; doi:10.1016/j.ctro.2024.100777)
Supplement: Supplementary data 1 [file mmc2.docx]

SUPPLEMENTARY MATERIALS:

Supplementary Table: General tumour characteristics: presence of metastasis at the time of diagnosis and primary tumour site, and related CSI doses depending on type of tumour.

According to the 2021 WHO Classification of Tumours of the CNS .

^*^Percentage of metastatic patients at diagnosis among the different types of tumours.

| **Type of tumour (WHO 2021)** | | **Total**  n (%) | **Metastasis at diagnosis**  n (%^*^) | **Primary tumour location** - n | | | **CSI Dose**  **in Gy** - n | | | | |
| --- | --- | --- | --- | --- | --- | --- | --- | --- | --- | --- | --- |
|  |  |  |  | **ST** | **PF** | **Med** | **18** | **23.4** | **24** | **30** | **36** |
| Embryonal tumours | | 57 (72.2%) | 21 (36.8%) | 3 | 53 | 1 | 5 | 16 | 0 | 0 | 36 |
| Medulloblastoma | | 53 (67.1%) | 17 (32.1%) | 0 | 53 | 0 | 4 | 16 | 0 | 0 | 33 |
| *Histologically*  *defined* | *Classic*  *Nodular / Desmoplastic*  *Anaplastic / Large cell*  *Missing* | *22 (27.8%)*  *20 (25.3%)*  *10 (12.7%)*  *1 (1.3%)* | *8 (36.4%)*  *5 (25.0%)*  *3 (30.0%)*  *1 (100%)* |  |  |  |  |  |  |  |  |
|  |  |  |  |  |  |  |  |  |  |  |  |
|  |  |  |  |  |  |  |  |  |  |  |  |
|  |  |  |  |  |  |  |  |  |  |  |  |
| *Molecularly*  *defined* | *WNT-activated*  *SHH-activated/TP53-wt*  *SHH-activated.TP53-mut*  *non-WNT/non-SHH* | *3 (3.8%)* | *1 (33.3%)*  *6 (37.5%)*  *0 (0%)*  *10 (32.3%)* |  |  |  |  |  |  |  |  |
|  |  | *16 (20.3%)* |  |  |  |  |  |  |  |  |  |
|  |  | *3 (3.8%)* |  |  |  |  |  |  |  |  |  |
|  |  | *31 (39.2%)* |  |  |  |  |  |  |  |  |  |
| ETAN-TR | | 2 (2.5%) | 2 (100%)  2 (100%) | 2 | 0 | 0 | 0 | 0 | 0 | 0 | 2 |
| PNET / CNS Embryonal tumour | | 2 (2.5%) |  | 1 | 0 | 1 | 1 | 0 | 0 | 0 | 1 |
| Germ cell tumours | | 8 (10.1%) | 6 (75.0%)  3 (75.0%)  2 (66.7%)  1 (100%) | 8 | 0 | 0 | 0 | 0 | 5 | 3 | 0 |
| Germinoma | | 4 (5.1%) |  | 4 | 0 | 0 | 0 | 0 | 4 | 0 | 0 |
| NGGCT | | 3 (3.8%) |  | 3 | 0 | 0 | 0 | 0 | 0 | 3 | 0 |
| Mixed germ cell tumour | | 1 (1.3%) |  | 1 | 0 | 0 | 0 | 0 | 1 | 0 | 0 |
| Choroid plexus tumours | | 5 (6.3%) | 5 (100%)  2 (100%)  3 (100%) | 5 | 0 | 0 | 0 | 0 | 0 | 0 | 5 |
| Atypical choroid plexus Papilloma | | 2 (2.5%) |  | 2 | 0 | 0 | 0 | 0 | 0 | 0 | 2 |
| Choroid plexus Carcinoma | | 3 (3.8%) |  | 3 | 0 | 0 | 0 | 0 | 0 | 0 | 3 |
| Gliomas and glioneuronal tumours | | 4 (5.1%) | 4 (100%)  1 (100%) | 3 | 1 | 0 | 0 | 0 | 0 | 0 | 4 |
| Astroblastoma, MN1-altered | | 1 (1.3%) |  | 1 | 0 | 0 | 0 | 0 | 0 | 0 | 1 |
| Diffuse glioneuronal tumour | | 2 (2.5%) | 2 (100%)  1 (100%) | 1 | 1 | 0 | 0 | 0 | 0 | 0 | 2 |
| Papillary glioneuronal tumour | | 1 (1.3%) |  | 1 | 0 | 0 | 0 | 0 | 0 | 0 | 1 |
| Ependymal tumours | | 4 (5.1%) | 3 (75.0%)  1 (50.0%)  2 (100%) | 0 | 2 | 2 | 0 | 0 | 0 | 0 | 4 |
| Posterior fossa ependymoma, PFA | | 2 (2.5%) |  | 0 | 2 | 0 | 0 | 0 | 0 | 0 | 2 |
| Spinal ependymoma | | 2 (2.5%) |  | 0 | 0 | 2 | 0 | 0 | 0 | 0 | 2 |
| Pineal tumours | | 1 (1.3%) | 0 (0%)  0 (0%) | 1 | 0 | 0 | 0 | 0 | 0 | 0 | 1 |
| Pinealoblastoma | | 1 (1.3%) |  | 1 | 0 | 0 | 0 | 0 | 0 | 0 | 1 |
| **Total** | | **79 (100%)** | **39 (49.4%)** | **20** | **56** | **3** | **5** | **16** | **5** | **3** | **50** |

Med= Medullar; NGCCT= Non-Germinomatous Germ-Cell Tumour; PF=Posterior Fossa; ST=Supratentorial
